# The Path from Nasal Tissue to Nasal Mucosa on Chip: Part 2—Advanced Microfluidic Nasal In Vitro Model for Drug Absorption Testing

**DOI:** 10.3390/pharmaceutics15102439

**Published:** 2023-10-09

**Authors:** Eugen Viktor Koch, Sebastian Bendas, Kristina Nehlsen, Tobias May, Stephan Reichl, Andreas Dietzel

**Affiliations:** 1 Institute of Microtechnology, TU Braunschweig, Alte Salzdahlumer Str. 203, 38124 Braunschweig, Germany; 2 Center of Pharmaceutical Engineering, Franz-Liszt Str. 35 a, 38106 Braunschweig, Germany; s.bendas@tu-braunschweig.de (S.B.);; 3 Institute of Pharmaceutical Technology and Biopharmaceutics, TU Braunschweig, Mendelssohnstr. 1, 38106 Braunschweig, Germany; 4 InSCREENeX GmbH, Inhoffenstr. 7, 38124 Braunschweig, Germany

**Keywords:** organ on chip, microfluidics, nasal mucosa, nasal drug administration, permeation test, mucociliary clearance

## Abstract

The nasal mucosa, being accessible and highly vascularized, opens up new opportunities for the systemic administration of drugs. However, there are several protective functions like the mucociliary clearance, a physiological barrier which represents is a difficult obstacle for drug candidates to overcome. For this reason, effective testing procedures are required in the preclinical phase of pharmaceutical development. Based on a recently reported immortalized porcine nasal epithelial cell line, we developed a test platform based on a tissue-compatible microfluidic chip. In this study, a biomimetic glass chip, which was equipped with a controlled bidirectional airflow to induce a physiologically relevant wall shear stress on the epithelial cell layer, was microfabricated. By developing a membrane transfer technique, the epithelial cell layer could be pre-cultivated in a static holder prior to cultivation in a microfluidic environment. The dynamic cultivation within the chip showed a homogenous distribution of the mucus film on top of the cell layer and a significant increase in cilia formation compared to the static cultivation condition. In addition, the recording of the ciliary transport mechanism by microparticle image velocimetry was successful. Using FITC-dextran 4000 as an example, it was shown that this nasal mucosa on a chip is suitable for permeation studies. The obtained permeation coefficient was in the range of values determined by means of other established in vitro and in vivo models. This novel nasal mucosa on chip could, in future, be automated and used as a substitute for animal testing.

## 1. Introduction

The nasal administration of systemic drugs offers many advantages over other routes of drug delivery, because of strong vascularization, the accessibility of the nasal cavity and the avoidance of the first-pass metabolism [1,2,3,4]. At the front line of defense of the respiratory system, the drug absorbance through the nasal mucosa is limited by the physical barrier of the epithelium, the mucus layer and the mucociliary clearance (MCC) [5,6,7].

Traditionally, animals, either as donors for excised tissue or as in vivo model, have been used to study the permeation mechanism of pharmaceutical compounds across the nasal mucosa [8,9]. Recently, 2D cell culture models have evolved into organ-on-chip platforms with 3D tissue-engineered scaffolds [10]. Various in vitro models of the nasal mucosa-on-chip (NM-chip) have been proposed, capable of stimulating the epithelial cells to differentiate in a physiological manner by the implementation of air exposure and mechanophysical stresses to form a confluent mucus layer or to enhance ciliary functionality to promote mucociliary transportability [11,12,13,14,15,16].

In combination with a suitable epithelial cell line, the NM-chips could be utilized to analyze the permeation mechanism in a physiologically relevant cellular microenvironment, whereby considerably reducing the necessity of animal experiments [17,18,19].

There is much interest in the established RPMI 2650 cell line, a carcinoma derived from a human nasal cell line capable of mucin secretion [11,20,21,22,23]. Due to the lack of cilia generation, and therefore formation of a functional MCC, the RPMI 2650 cell line is only conditionally suitable to generate a complex nasal cell model. Thus, primary nasal epithelial cells, from human or animal origin, are still mostly used to form more complex models of the nasal mucosa [24,25,26,27]. Once isolated and well differentiated, these cells initially express all the properties required for a permeation model, but lose their abilities to form tight junctions within the next subcultures [28]. Commercially available models of human nasal epithelial cells like MucilAir™, developed by Epithelix, require relatively long cultivation times for differentiation before permeation studies can be performed [29].

Hence, part 1 of our work describes a new immortalized respiratory epithelial cell line P1, derived from pig’s snouts, developed and characterized in a long-term cultivation period (>30 days), cultured at the air–liquid interface (ALI). The cell line P1 differentiated and expressed cilia and tight junctions, and formed an adequate amount of mucus. This so called ANaMuc model has been utilized for permeation studies, showing promising results for the permeation of FITC-dextran 4000 [30].

Here in part 2, we developed and implemented an advanced microfluidic-based nasal in vitro model for drug permeation studies. This so-called NM-chip is designed to mimic the airstream condition of the human nasal cavity during breathing, in order to stimulate the cell layer by a physiological wall shear stress (WSS). As with the previously published tissue-barrier-on-chip platform, cells shall be pre-cultivated on a polymer membrane in a static holder before being transferred to the chip for subsequent dynamic cultivation [31]. The chip shall allow study of the effect of WSS on the production of mucus, formation of cilia, mucociliary transport, TEER (Transepithelial Electrical Resistance) and permeation of substances.

## 2. Materials and Methods

### 2.1. NM-Chip Design

**Figure 1:**
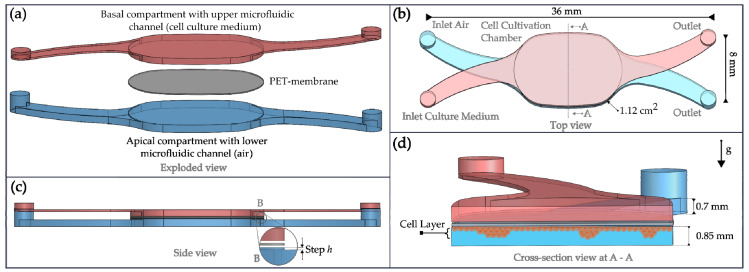
Fluidic design of the NM-chip. (**a**) Exploded view showing a basal compartment for continous cell culture medium supply, a porous PET membrane interlayer and an apical compartment to guide the periodic airflow. (**b**) Top view with geometrical dimensions of the cell cultivation chamber in the center together with inlets and outlets. (**c**) Side view of the chip design with a detail view of the transition with a step *h* between membrane surface and apical inlet channel roof. (**d**) Cross-section of the cell cultivation chamber with upside-down hanging P1 cell layer (g indicates the direction of gravity).

The NM-chip, as illustrated in Figure 1a, was designed with two identical compartments. The basal compartment was intended for the transport of cell culture media and the apical compartment for the periodic airflow. In the center of the chip, the cell cultivation chamber connects both compartments via a porous membrane made of polyethylene terephthalate (PET), as shown in Figure 1b. The cell cultivation chamber covers an area of 1.12 cm^2^, equivalent to the membrane area of a 12-well Transwell^®^ (Hong Kong, China) Insert.

In the apical compartment, the inlet and outlet channels were made shallower than the cell cultivation chamber (see Figure 1c) to create a homogenous tangential airflow above the cell layer. The influence of the step height *h* was investigated by FEM simulations. As illustrated in Figure 1d, during dynamic cultivation, the cells could be cultivated upside down in the apical compartment and continuously supplied with cell culture media through the porous membrane above.

### 2.2. Computational Fluid Dynamics

The computational fluid dynamics (CFD) simulation of the microfluidic channel was conducted using the Fluent module in the software ANSYS 2020 R1 (ANSYS, Canonsburg, PA, USA). A mesh was generated with an element size of 40 µm, composed of tetrahedral (Tet10) and prism wedge elements (Wed15). The prism layer was placed on the bottom of the cell cultivation chamber to provide sufficient scope to reveal detailed flow characteristics and resolution of the wall shear stress. The quality of the mesh had an average skewness of 0.22 with SD of 0.12 and an aspect ratio of 1.93 with SD of 0.68, which was comparable to CFD simulations of nasal irrigation flow as described in the literature [32].

No-slip boundary conditions at the walls and a zero outlet pressure were assumed. The properties for air were adapted for a temperature of 37 °C. The segregated solver SIMPLEC algorithm was implemented to couple the pressure and velocity fields, and a second-order upwind scheme was used for discretization. For the velocity inlet, a sine wave function, representing inhalation and complete reversed airflow during exhalation, was defined with a peak-to-peak flowrate of 110 mL/min and a breathing cycle of 6.5 s. For the calculation, the time step size was set to 0.2 s for a time period of a breathing cycle, the residuals were set to 0.5 × 10^−5^.

### 2.3. Microfabrication of the Chip

**Figure 2:**
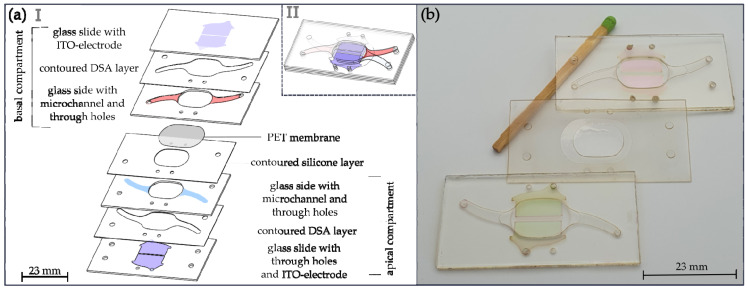
The microfabricated NM-chip. (**a**) (I) Exploded view illustrating the sandwich construction with eight layers. The basal compartment consists of (from top to bottom): a glass slide with electrodes made of ITO; a contoured layer of DSA; and a glass slide layer with microchannel, through-holes and the cell cultivation chamber in the center. The apical compartment consists of (from bottom to top): a glass slide with ITO counter electrodes, vias and through-holes; a contoured DSA layer; and a glass slide layer with microchannel, through-holes and the cell cultivation chamber in the center. The basal and apical system halves are connected by the contoured silicone layer with through-holes and the contoured PET nanoporous membrane placed in the center. (II) Sketch of the assembled chip. (**b**) Photograph of the NM-chip system halves with the contoured silicone layer with PET membrane.

The chip, as shown in Figure 2a, was fabricated in a sandwich structure made of glass (BOROFLOAT^®^ 33 with 0.7 mm in thickness, Schott, Mainz, Germany), double-sided adhesive tape (ARCare^®^, 0.15 mm in thickness, ADHESIVE RESEARCH, Glen Rock, PA, USA) [33], a porous PET membrane (11 µm in thickness with a pore size of 0.4 µm and a pore density of 2.1 × 10^6^ pores/cm^2^, Sabeu, Northeim, Germany) and a thin silicone layer (GP 60, MVQ silicones, Weinheim, Germany), which provides leakage-free operation but also allows the opening of the chip at any time.

The structuring of the microfluidic channels, vias and contours were realized by femtosecond laser (fs-laser) ablation using a laser workstation (Microstruct-C, 3D–Micromac, Chemnitz, Germany). The system was equipped with a Yb:KGW (Ytterbium-doped potassium–gadolinium–tungstate) femtosecond laser source (Pharos, Light Conversion, Vilnius, Lithuania) emitting at the fundamental wavelength of 1030 nm with a pulse duration of 212 fs. The deflection of the laser beam was controlled with a galvanometer scanner (Scanlab RTC5, Puchheim, Germany). The beam was focused on the surface by an f-theta lens with a 100 mm focal length to a spot of around 20 µm in diameter exhibiting a Gaussian intensity distribution profile.

Laser parameters for minimizing material damages like crack formation and burning artefacts had been evaluated in advance. The microfluidic channels in glass were micromachined at pulse intensities of 11.85 µJ at a laser writing speed of 1500 mm/s; for the vias, a higher pulse energy of 84.6 µJ and a slower laser writing speed of 600 mm/s were necessary to remove bulk material. The double-sided adhesive (DSA) layer was contoured applying pulse intensities of 10.02 µJ and a writing speed of 1500 mm/s, and vias in the silicone rubber were made with 101.4 µJ and a writing speed of 1500 mm/s. The porous cell cultivation PET membranes were cut to the shape of the cell cultivation chamber, but with an additional margin of 2 mm, using pulse energies of 0.88 µJ and 1500 mm/s using frequency-doubled emission at 515 nm. After fs-laser structuring, all layers were cleaned in an ultrasonic bath for 10 min in 50% ethanol in DI-water.

The outer top and bottom glass slides were equipped with micro-electrode structures made of ITO (Indium–Tin–Oxide) to enable in situ TEER (Transepithelial Electrical Resistance) measurement in a tetrapolar configuration. Due to the relatively large electrode patterns, a shadow mask was fabricated using a thermosensitive adhesive foil (DICING TAPE VGF-07, Minitron Elektronik, Ingolstadt, Germany). The foil was also contoured by fs-laser ablation at pulse energies of 4.82 µJ and a writing speed of 1500 mm/s. The foil was then aligned and taped on the glass layer to deposit a thin ITO layer by magnetron sputtering using argon as process gas at a power of 100 W at the ITO target (LS 440 S, Ardenne Anlagentechnik, Dresden, Germany). Afterwards, the thermosensitive foil was completely removed by heating the wafer at 140 °C for 5 min, leaving rectangular ITO micro-electrodes with an area of 46 mm^2^ behind. The vias in the bottom layer were filled with a conductive glue (DUALBOND IC343, DELO, Windach, Germany). To improve wettability and increase adhesion of the conductive glue, the wafer was treated by an oxygen plasma for 2 min with O_2_ (60 sccm) at 150 W in a barrel etcher (308 PC Barrel Etching System, STS Surface Technology Systems, Yvonand, Switzerland).

The two glass layers of the respective compartments were joined by means of the DSA layers, as illustrated in Figure 2b. The basal and apical compartments were finally connected with a contoured silicone layer as a seal.

### 2.4. Membrane Transfer Tooling and Chip Mount

**Figure 3:**
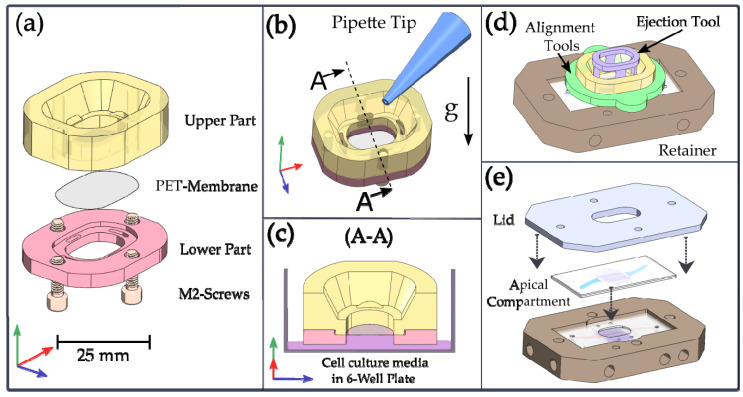
Technical drawings of the devices for membrane transfer and chip mounting. (**a**) Exploded view of the transfer holder, showing the upper and lower part of the transfer tooling with the membrane in between. (**b**) Chip in transfer holder ready for cell seeding using a pipette. (**c**) Assembled transfer holder with cell culture media wetting in the apical compartment. (**d**) Membrane aligned on the apical chip compartment and ready for transfer using the ejection tool. (**e**) Closed NM-chip within the chip mount ready for dynamic cultivation and permeation experiments.

As shown in Figure 3a, a transfer holder was designed consisting of an upper and a lower part so that the nanoporous PET membrane could be clamped in between by four screws. Both parts were made of polyetheretherketone (PEEK) by precision CNC machining. The parts were coated with hydrophobic parylene C by CVD (SMÅLAND, NTTF coatings, Rheinbreitbach, Germany) to avoid the growing of cells when cells were injected through a pipette as illustrated in Figure 3b. The cavity that forms underneath the membrane can be carefully filled with cell culture media, which is kept in place by capillary forces, as depicted in Figure 3c, allowing a media level that is slightly higher than in the surrounding volume of a six-well plate, thereby avoiding static pressure on the cells through the membrane. An ejection tool for the release of the membrane and an alignment tool to replace the lower part of the transfer holder after static cell cultivation were realized by 3D printing (AR-M2 material, AGILISTA-3200W, Keyence Germany, Neu-Isenburg, Germany). These parts allowed for the alignment of the membrane on the chip’s apical compartment, covered with the contoured silicone layer which was already placed in the chip mount, as depicted in Figure 3d. The chip mount could be closed with a metal lid and four M4 metal screws with compression springs underneath (Febrotec Federn, Halver, Germany) after the membrane had been transferred and the basal chip compartment had been added, as depicted in Figure 3e. Besides the ejection and alignment tools, which can be sterilized in a 70% (*v*/*v*) ethanol in DI-water, all other parts can be autoclaved. The transfer procedure was also recorded and can be found in the Supplementary Material.

### 2.5. Cell Cultivation

Due to the relatively long cultivation time required for differentiation of the ANaMuc model, and also to allow parallelization of cell culture experiments, P1 cells were seeded and cultivated in the transfer holder under static conditions according to the protocol previously described in detail [30]. After 7, 10 and 14 days, the membranes were transferred to the chip system for 3 h to study the effect of periodic airflow-induced wall shear stress on the formation of mucus and cilia. Cells cultured for the same period under static conditions in transfer holders served as reference.

Prior to cell seeding with a density of 0.5 × 10^6^ cells per transfer holder (1.12 cm^2^), PET membranes were coated with rat tail collagen solution (1.5 mg/mL dissolved in ethanol and 0.05% acetic acid). Cells were cultured submerged (LCC) for one day. At day 2 of culture, after reaching confluence, cells were lifted to the air–liquid interface (ALI), followed by 7 to 14 days of cultivation. The cell culture medium (HuNASA from InSCREENeX, Braunschweig, Germany) was changed three times a week.

### 2.6. Experimental Setup

**Figure 4:**
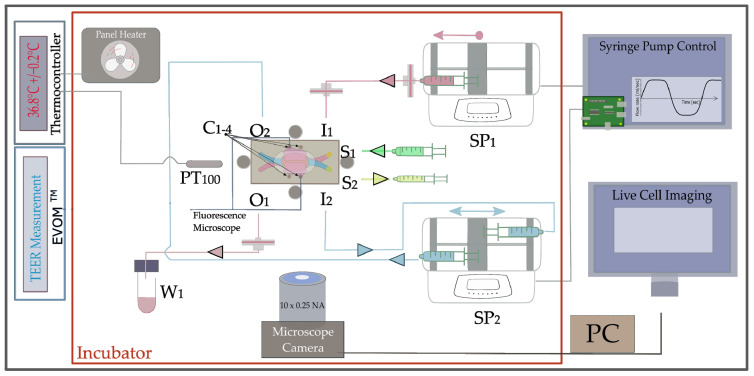
Schematic view of the experimental setup. The mounting bracket holding the NM-chip was equipped with inlets I1 and I2, outlets O1 and O2, side ports S1 and S2 and contact holes C1-4 to the ITO-microelectrodes. The cell culture medium was pumped by syringe pump SP1 through the chip and collected in the waste reservoir W1 as indicated by the red arrows. The periodic bidirectional airflow indicated by the blue arrows was generated by the opposing syringes in syringe pump SP2, which was microprocessor-controlled. The chip mounting bracket was positioned on top of an inverted fluorescence microscope with a camera. The setup was placed in a self-built incubator (red frame) in which the temperature was stabilized by a panel heater that was controlled by a thermo-controller coupled to a Pt-100 temperature sensor. The TEER value could be read out using EVOM^2®^ electronics connected to the ports C1-4. The ports S1 and S2 were only opened while drawing fluid samples (yellow arrow) and replenishing media (green arrow) for permeation analysis.

The experimental setup, as illustrated in Figure 4, consisted of the microfluidic mounting bracket containing the NM-chip, two syringe pumps SP1 (KDS series 200, KD Scientific, Holliston, MA, USA) and SP2 (KDS series 260, KD Scientific) and an inverted microscope (Primovert, Zeiss, Oberkochen, Germany). With the exception of the electronic control and evaluation units, all components were placed in a self-built incubator made of PMMA (polymethylmethacrylat) in which a temperature of 36.8 ± 0.2 °C was stabilized by a panel heater (SH130, Elmeko, Liebenscheid, Germany) controlled by a thermo controller (Jumo di eco, Jumo & Co., Fulda, Germany) coupled to an integrated thermosensor PT100. The microscope was equipped with a 10× plan achromatic objective and a microscope camera (PowerPack ace 3.2MP, Basler, Ahrensburg, Germany), allowing continuous monitoring of the cell layer. The mounting bracket holding the microfluidic chip had two inlets (I1, I2) and two outlets (O1, O2) which led into the microchannel. Two additional side ports (S1, S2) at both inlets allowed first, evacuation of trapped air in the tubings while filling the chip with cell culture medium, and second, the introduction of donor fluid (into the apical compartment) and the drawing out of acceptor fluid (from the basal compartment) volumes during permeation experiments. Syringe pumps were connected via PTFE tubes (ID = 0.750 mm, Techlab, Braunschweig, Germany) using flangeless fittings made of Delrin (H&S P-307, IDEX, Lake Forest, IL, USA). To avoid contamination inside the chip, the tubing for the cell culture medium was interrupted by hydrophilic sterile syringe filters (PTFE, 0.2 µm, Whatman, Maidstone, UK). Cell culture medium was supplied by syringe pump SP1 equipped with a 5 mL plastic syringe connected to I1 and was collected after having passed the chip through O1 in the waste reservoir W1. Syringe pump SP2 held two opposing 100 mL plastic syringes filled with sterile air to generate the periodic bidirectional airflow through the apical chip compartment via I2 and O2. The simultaneous push and pull by SP2 avoided unfavorable pressures acting on the cells. SP2 was controlled by a microprocessor (Raspberry Pi 4, Raspberry Foundation, Cambridge, UK) coupled to a touch display (7 inch IPS display, Jun-Saxifragelec, purchased via Amazon Germany, Munich, Germany). For the experiments, breathing cycles with a flow rate of 110 mL/s for 3 s inhalation and 3 s exhalation, with a 3 s pause in between, were programmed. Respiratory phases of 132 s alternated with rest phases of the same duration. Prior to the experiments, an EVOM2 ^®^ resistance meter (World Precision Instruments, Sarasota, FL, USA) was connected to the ports C1–C4 using contact pins. To determine the TEER values, both chip compartments were filled with cell culture medium. Afterwards, the cell culture medium was carefully removed from the apical compartment.

### 2.7. Mucin Staining

After the experiment, the chip was disassembled and the membrane was extracted for mucin staining with alcian blue following an established protocol [34]. First, cells were washed with PBS and fixed with 4% PFA (paraformaldehyde) for 10 min. Then, cells were rinsed with PBS and 3% acetic acid (*v*/*v*). Subsequently, 1% alcian blue (*v*/*v*) (Sigma-Aldrich Chemie, Taufkirchen, Germany) was added to the apical cell layer for 30 min. Afterwards, two rinsing steps were performed. Counterstaining of the cell nuclei was performed with nuclear fast red-aluminum sulfate solution (Carl Roth, Karlsruhe, Germany) for 4 min. The stained samples were analysed by means of a 3D digital microscope (VHX-5000, Keyence Germany, Neu-Isenburg, Germany). To determine the relative area covered with mucus, 10 images with a magnification of 400 were randomly taken and analyzed, applying a bandpass hue color filter in the range of 145–188 and a saturation filter of 140 to 255 to conservatively extract only intense blue-stained areas. Additionally, the apical compartment of the NM-chip was stained with alcian blue to detect mucus entering the channels and spreading on the bottom of the cultivation chamber.

### 2.8. Immunofluorescence Staining of Cilia

Membrane samples extracted from the chip were washed with PBS and fixed with PFA (paraformaldehyde) for 10 min. Subsequently, the cells were permeabilized with 0.1% Triton X in PBS for 10 min, followed by three washing steps with PBS and blocking with 10% normal goat serum (Vector laboratories, Burlingame, CA, USA) in PBST (PBS with 0.1% Tween 20) for 45 min at room temperature. The anti-γ-tubulin antibody (ab 27074, Abcam plc, Cambridge, UK) was diluted 1:500 with 0.1% BSA in TBST (Tris buffered saline with Tween 20) and incubated overnight at 4 °C. Afterwards, cells were rinsed with PBS three times for 5 min and incubated with an anti-Mouse IgG H&L Alexa Fluor 568 antibody (ab 175473, Abcam plc, Cambridge, UK), diluted in PBS (1:400), for 1 h at room temperature. The cell nuclei were counterstained with Hoechst 33342 (Sigma-Aldrich Chemie, Taufkirchen, Germany) 1:50 in PBS (stock solution 1 mg/mL in 50% ethanol) for 15 min at room temperature and light protection, followed by two washing steps with PBS. Samples were imaged using an inverted epifluorescence microscope IX73 (Olympus, Tokyo, Japan) equipped with CellSens imaging software 1.18. The quantitative evaluation of the fluorescence measurements of red- and blue-stained regions was performed by a machine learning segmentation algorithm using the WEKA segmentation plugin of Fiji software with subsequent area fraction analysis of the disaggregated colors [35,36].

### 2.9. PIV Analysis

In order to recognize the beating of the cilia and to evaluate the transport mechanism of the mucus, microscope movies from the cell layer within the chip were recorded during the experiment, right after installing the microsystem in the setup, for 10 s, applying a frame rate of 25 ms; during the three-hour experiment, a frame was taken every 3 s and at the end once more, for 10 s with a frame rate of 25 ms. To evaluate the mucus movement, a sequence, during resting mode, of at least 40 frames was taken from a cultivation time of 2.5 h, and a PIV algorithm was applied using PIVlab software (v2.58) [37], whereby dead cells and round-shaped mucin fragments were traced. The FFT window deformation algorithm was applied with a high correlation robustness; three passes were chosen with an interrogation area of 200, 150 and 100 px, with a 50% shift step to cover at least three round tracer objects.

### 2.10. Permeation Experiments

FITC-dextran 4000 (mol wt 3.000–5.000, Sigma-Aldrich Chemie, Taufkirchen, Germany) with a concentration of 2500 µg/mL was used as marker substance with a molecular weight comparable to some peptide agents like glucagon (3.483 kDa) and calcitonin (3.418 kDa) administered by nasal spray. ANaMuc models within the NM-chip were washed once with prewarmed KRB (Krebs Ringer Buffer) and equilibrated for 30 min in KRB at 37 °C. Then KRB was removed and replaced basolateral (basal compartment, as shown in Figure 1) with 380 µL acceptor buffer (KRB). On the apical side (apical compartment, as shown in Figure 1), 470 µL of the test solution was applied. Samples of 380 µL were taken from the acceptor (basal compartment) after 10, 20, 30, 45, 60, 90, 120, 150 and 180 min, and replaced with the same volume of warmed KRB. The marker substance was analysed using a Tecan GENios fluorescence plate reader and Magellan software (Tecan, Männedorf, Switzerland). FITC-dextran 4000 was excited at 485 nm, and fluorescence was detected at 535 nm. The permeation coefficient *P_app_* was calculated according to Equation (1):

(1) $$Papp=\frac{\mathrm{dQ}}{\mathrm{dt}\cdot c_{0}\cdot A}$$

where *dQ*/*dt* is the flux (µg/s) of the respective substance across the barrier, $c_{0}$ is the initial donor concentration (µg/mL) and *A* is the membrane area (cm^2^). The experiment was performed in triplicate.

### 2.11. Statistical Analysis

The results were statistically analyzed for normality using Shapiro–Wilk and Kolmogorov–Smirnov tests. In the case of an evident normal distribution, a two-tailed, two-sample F test was performed followed by a two-tailed, two-sample *t* test using SPSS software 28 (IBM, Armonk, NY, USA). Moreover, *p* values less than 0.05 (* *p* < 0.05, ** *p* < 0.01, *** *p* < 0.001) were considered statistically significant.

## 3. Results and Discussion

### 3.1. Numerical Simulation

**Figure 5:**
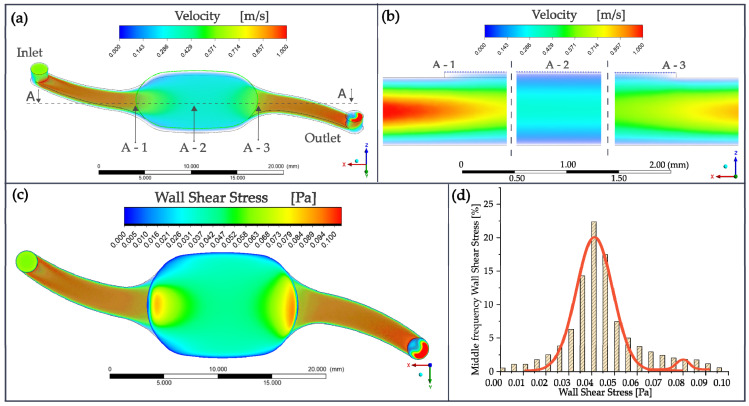
Simulation of the airflow inside the NM-chip at 110 mL/min. (**a**) Velocity profile at medium channel height with the airflow direction from left to right at a slightly tilted view. (**b**) Airflow velocity cross-sectional profiles at the inflow (A-1), in the center (A-2) and at the outflow of the chamber (A-3). (**c**) Airflow distribution of resulting WSS (top view) at the bottom of the cell cultivation chamber. (**d**) Histogram showing the distribution of the WSS (and indication of underlying Gaussian distributions) in the cell cultivation chamber during inhalation.

Figure 5a shows the air velocity profile at medium channel height in the apical compartment obtained by CFD simulation in the NM-chip model at a flow rate of 110 mL/min. In the narrow inlet and outlet channels, the velocity exceeded 1 m/s. The velocity inside the cell cultivation chamber was rather homogenous, around 0.3 m/s, with the exception of two areas of higher magnitude in the vicinity of the inlet and outlet channels. These transition areas, as well as the center of the cultivation chamber, are shown in Figure 5b in cross-sectional view. To investigate the influence of the tissue grown on the membrane, the airflow-induced WSS distribution on the cell layer is shown in Figure 5c. Similarly to the velocity profile, two WSS peaks around 0.1 Pa arose at the transition area in an otherwise quite homogenous field. As shown by the histogram in Figure 5d, the distribution of the WSS in the cell cultivation chamber almost followed a superposition of two Gaussian distributions, a major one around a WSS of 0.041 Pa and a minor one around 0.085 Pa, representing the transition areas. This results corresponds to numerical simulations of the human nasal cavity based on X-ray CT (computer tomography) scans of 25 healthy noses at a steady airstream of 200 mL/min. A WSS of around of 0.045 Pa for around 15% of the nasal surface and 0.1 Pa for around 3% were found during inspiration [38]. Similar wall shear stresses and velocities for the human nasal cavity were obtained from a numerical analysis of the respiratory pathway [39,40,41,42,43,44].

### 3.2. TEER Measurements

The TEER values from the NM-chip obtained after 7, 10 and 14 days are listed in Table 1. These values follow the same trend as measured in Transwell^®^ Inserts in a static environment, but they were noted as being slightly higher. Further, the cell culture media filling and emptying procedure in the apical compartment, which was necessary to perform TEER measurements, resulting in the washing off of mucus from the cell layer, as detected by mucin staining with alcian blue. Therefore, after TEER measurements, the samples were not taken in further on-chip experiments. The measured TEER values ranged between literature TEER values obtained from human nasal primary epithelial cells after 21 days of ALI cultivation (600–850 Ω∙cm^2^) [24] and values from the carcinoma-derived nasal cell line RPMI 2650 (80–120 Ω∙cm^2^) [20].

### 3.3. Mucus Production

**Figure 6:**
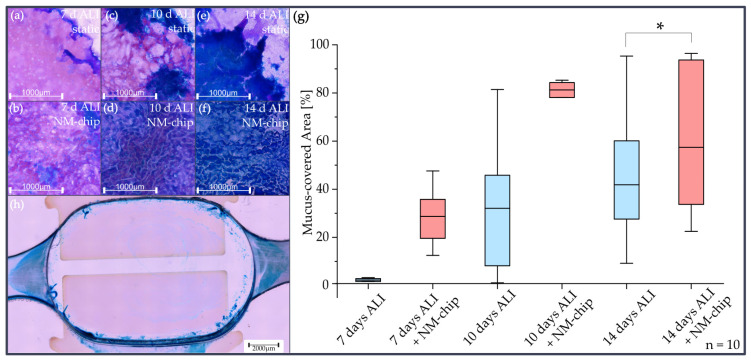
Confocal microscopy bright field images of alcian blue staining of mucus, performed at time stages 7 (**a**,**b**), 10 (**c**,**d**) and 14 (**e**,**f**) days of static cultivation at ALI in the transfer holder with membrane, and for each case after subsequent dynamic cultivation with periodic airflow for 3 h in the NM-chip. (**g**) The area fraction covered with mucus was obtained by image analysis for the same cultivation conditions (n = 10). The center lines in the box plots represent the median, the outer box edges show the 25% and 75% percentiles, and the whiskers show the outliers. The larger variations after 14 days result from small uncovered islands (* indicates *p* < 0.05). (**h**) NM-chip apical compartment (after membrane removal) with mucus stained from cell culture after 7 days ALI with NM-chip cultivation.

Confocal microscopy images of the membrane, as shown in Figure 6, illustrate the progressive production of mucus in the different differentiation stages of the ANaMuc model. It was cultivated statically in the transfer holder followed by three hours of dynamic cultivation in the NM-chip. After 7 days of static cultivation, mucus was detected only at the edge of the cell layer, as shown in Figure 6a. The staining of nuclei shows a rather homogenous cell layer. Only with a subsequent cultivation in the NM-chip, as shown in Figure 6b, were areas of mucus found not only at the edge of, but also on top of, the cell layer. Even after 10 days of static cultivation, a similar mucus and nuclei staining pattern was discovered after 7 days. However, with additional NM-chip cultivation, a homogenously distributed mucus film was formed, as shown in Figure 6d. After 14 days, the short additional dynamic on-chip cultivation expressed a thicker mucus film on top of the cell layer, as can be seen from the intense blue color in Figure 6f. Although the thickness of the mucus film increased, small spots remained uncovered. Without the dynamic cultivation, large mucus lumps formed on top of the cell layer, as shown in Figure 6e. Figure 6g reflects these facts by the quantitative image analysis, in which the area fraction covered with mucus was determined. In Figure 6h, the apical compartment of the chip after 7 days of ALI cultivation with NM-chip is shown after removal of the membrane. The stained mucus could be detected at the edge of the cell cultivation chamber and partly inside the inlet and outlet channel.

With the mechanophysical stimulation within the chip, the mucus formation of all cell cultures increased drastically compared with the static reference, between a factor of 15 for the cell culture after 7 days, a factor of 2.5 for the cell culture after 10 days and a significant increase for the cell culture after 14 days.

These results are in accordance with the literature, where the increase in mucin secretion was around 30–80%, as obtained with primary human nasal epithelial cells, after a WSS of 0.05 Pa and 0.5 Pa respectively, for a time period of 5 min, as was previously described in [45]. Furthermore, the influence of mechanophysical stimulation on mucus secretion was reported to be affected more strongly by the time length of WSS than by the level of WSS [46]. With the RPMI 2650 cell line, as was observed here after 14 days, a similar intense alcian blue staining of the mucus film did not result before 21 days of culturing at an applied WSS of 0.004 Pa [11,47].

### 3.4. Formation of Cilia

**Figure 7:**
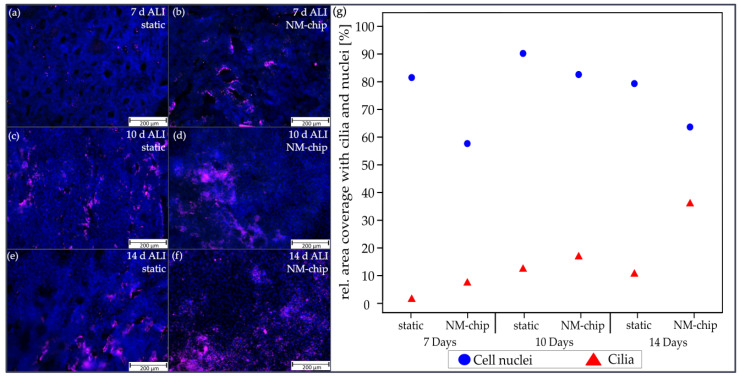
Immunohistochemical fluorescence staining of P1 cells by antibody staining (red) for γ-tubulin as evidence for ciliated- cells combined with cell nuclei staining (blue) with Hoechst 33342. Fluorescence images of P1 cells after (**a**) 7, (**c**) 10 and (**e**) 14 days in static ALI cultivation in the transfer holder and (**b**,**d**,**f**) with additional short dynamic cultivation of 3 h in the NM-chip in each case. (**g**) Area fractions with cilia (red) and cell nuclei (blue), which were determined via image processing for all cases considered (n = 1).

Figure 7 shows the fluorescence images with immunofluorescence staining against γ-tubulin, as the indicator for ciliated cells, at different differentiation stages after static cultivation, and, in each case, with three hours of subsequent dynamic cultivation in the NM-chip.

After 7 days, first small areas of cilia could be detected, as shown in Figure 7a. With subsequent on-chip cultivation, morphological changes towards a more spongy-like texture appeared, as shown in Figure 7b. Here, the ciliated cells were detected mostly around openings in the cell layer. After 10 days, the cell layer became more homogenous, as shown in Figure 7c. The area fraction with cell nuclei and for cilia increased, but with NM-chip dynamic cultivation, the area of stained cell nuclei reduced but the area for cilia increased, as shown in Figure 7d. After 14 days of static cultivation, the detected area fraction of ciliated epithelial cells did not change compared to the equivalent sample after 10 days, as shown in Figure 7e. However, Figure 7f shows that with NM-chip cultivation, larger continuous fields with cilia were found. Furthermore, the cell nuclei staining indicates areas of lower cell densities. As can be seen in the quantitative analysis given in Figure 7g, cell densities generally decreased after 10 days. With additional cultivation on the chip, the cell density decreased slightly in each case, but cilia formation could be still promoted.

A similar effect of applied airflow on cilia formation has already been demonstrated in human tracheal epithelial cells. The amount of cilia formed from cells cultured for 35 days in a static compartment at ALI was identical to that of cultivations that were conducted for over 24 days in static conditions, and which were also followed by 24 h of dynamic cultivation at 0.05 Pa WSS [48].

### 3.5. Mucociliary Transport

**Figure 8:**
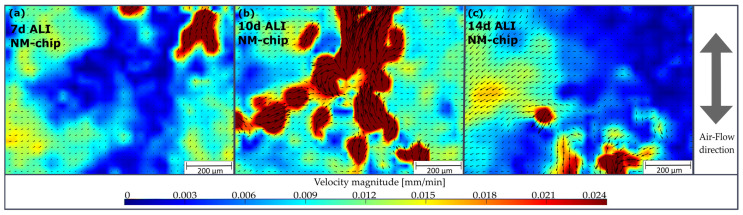
Vector fields (arrows indicate direction and magnitude of local velocity) together with color-coded velocity magnitude obtained by PIV analysis from the time lapse recordings of an ANaMuc model cell layer within the NM-chip at (**a**) 7 days, (**b**) 10 days and (**c**) 14 days of static cultivation, followed by 3 h of dynamic cultivation on-chip in each case.

In Figure 8, the velocity vector fields obtained by PIV (the movies used are accessible in the Supplementary Material) tracked dead cells and round mucus fragments that moved over the cell layer. These are presented for the three cell differentiation stages in each case, which were followed by 3 h of on-chip dynamic cultivation. After 7 days of ALI cultivation, the vector field, as shown in Figure 8a, indicates velocity magnitudes up to 0.024 mm/min, whereby a uniform direction was found in only a small part of the area. After 10 days, the cilia became more agile, leading to a higher areal fraction exceeding velocities of 0.024 mm/min, as shown in Figure 8b. The direction of ciliary transport predominantly corresponds to the direction of airflow, with the exception of a local vortex that was formed in the center.

According to Figure 8c, the mucociliary transport after 14 days appeared weakened. As can be seen in the videos in the Supplementary Material, the decrease in mucociliary transport velocity was as a result of accumulated mucus in the upper region, which increases transport volumes but decreases transport velocity. The maximum velocity of 0.024 mm/min that was measured by PIV (tracking fragments with sizes of 5–10 µm were found) is lower than the value obtained with human tracheal epithelial cells (HTEC) using fluorescence beads (which were 0.5 µm in diameter) [48]. In this work, the cells were cultured for 24 days, and then subsequently placed for 24 h in an airflow system, before the mucus was completely washed off and a bead mean velocity of 1.50 ± 0.26 mm/min was measured. For static cell cultures, higher bead velocities that range from 2.4–5.4 mm/min were reported [49]. An investigation of the MCC of 20 non-smoking adults, which was performed using a roentgenographic method with nasal injected reflective discs that had a diameter of 1 mm, showed a mean velocity of 10.8 ± 3.2 mm/min [50].

We speculate that the discrepancy in mucociliary transport velocities results mainly from the lack of a continuous cell layer with differentiated cilia, as shown in Figure 7g. The formation of a vortex, as shown in Figure 8b, illustrates that the cilia beat is not sufficiently coordinated yet, which is necessary for ensuring an effective MCC [49].

### 3.6. Permeation Coefficient for FITC-Dextran 4000

In order to evaluate the feasibility of the NM-chip for drug permeation studies of high-molecular-weight substances, the permeation coefficient was determined using FITC-dextran 4000. Table 2 summarizes the permeation coefficients of the different investigated ANaMuc models and provides an overview of comparative values previously reported in the literature. First, after 14 days of ALI cultivation without an applied periodic airflow, the mean permeation coefficient (P_app_) in the NM-chip obtained from the two experiments was 4.366 ± 0.645 × 10^−7^ cm/s. The permeation coefficient of three identical experiments, after 14 days of ALI cultivation with a subsequent periodic airstream for 3 h, was 3.419 ± 0.499 × 10^−7^ cm/s. No significant difference was found with respect to the determined P_app_-values between the different experimental runs in the NM-chip. A negative effect of the applied wall shear stress on the epithelial integrity of the cell layer could thus be excluded.

In comparison with the ANaMuc model cultured at static conditions in Transwell^®^ inserts, the permeation coefficient for FITC-dextran 4000 was significantly lower in the NM-chip with an applied airflow. A reasonable explanation for the different permeation coefficient derives from the discrepancy of TEER values between NM-chip and Transwell^®^ inserts, which result from the slightly modified condition of cultivation in the transfer holder. The TEER values of 556 ± 72 Ω∙cm^2^ were measured in the NM-chip compared with 412 ± 39 Ω∙cm^2^ in the Transwell^®^ inserts. High TEER values represent a strong paracellular barrier of the tissue and impede the permeation of the hydrophilic marker substance FITC-dextran 4000 via this route. Thus, the lower TEER values in the static permeation studies resulted in higher permeation coefficients. The impact of the TEER value on the permeation coefficient of FD4 (fluorescein isothiocyanate dextran with an average molecular weight of 4000 Da) on mucus-secreting airway cell lines has been reported by Lee et al. [51].

Compared with the permeability of excised nasal mucosa from sheep and rabbit, as well as RPMI 2650 cells, the dynamic ANaMuc model in the NM-chip showed a high consistency with respect to the permeation properties of FITC-dextran 4000. Compared with the static and dynamic ANaMuc model, a significantly lower permeation coefficient was determined for MucilAir™ in our own investigations [30]. A plausible cause of this observation is the higher TEER values measured for the MucilAir™ constructs. However, as is also the case for human nasal primary cells, the investigations of Werner and Kissel showed a lower agreement in the P_app_ values with the results of excised nasal mucosa than was found with the ANaMuc models. Thus, with respect to the barrier properties, the dynamic ANaMuc model is a good alternative due to the high comparability of permeation coefficients to excised nasal tissue.

**Table 2:** Summary of permeation coefficients (10^−7^ cm/s) of FITC-dextran 4000 for the ANaMuc model in the NM-chip and static ANaMuc model in Transwell^®^ inserts. Mean ± SD, n = 2–6.

Nasal Mucosa Model	FITC-Dextran 4000
ANaMuc Transwell insert	5.13 ± 0.76 ^A^
NM-Chip 14 d static	4.366 ± 0.645
NM-Chip 14 d dynamic	3.419 ± 0.499
MucilAir™	0.28 ± 0.04 ^A^
Human nasal primary cells	1.8 ± 0.4 ^B^
RPMI 2650	6.9 ± 0.4 ^C^
Excised nasal mucosa	4.6 (sheep) ^D^ 5.2–12.75 (rabbit) ^E^

Ref. ^A^ [30], ^B^ [52], ^C^ [17], ^D^ [53], ^E^ [54,55]. Comparison with P_app_ values of the same marker compound determined using animal ex vivo models, human nasal primary cells and RPMI 2650 cell line reported in the literature.

## 4. Conclusions

Based on the immortalized nasal epithelial cell line presented in part 1, we established an on-chip model that allowed a dynamic cultivation of ANaMuc. In contrast to other microfluidic models of the human airway, a transfer concept was developed that facilitated the external cell pre-cultivation and integrated TEER measurement.

By applying a periodic bidirectional airflow, a physiological WSS was generated on the cell layer, thus representing the conditions found in the human nasal mucosa. This mechanophysical stimulus led to a significant increase in the mucus secretion and formation of motile cilia with still limited ability of mucociliary transport. Compared to the static ANaMuc model, the cell layer cultured under dynamic on-chip conditions builds up a more homogenous mucus film and expresses organotypic barrier properties that are similar to those of the nasal mucosa, as demonstrated by the permeation coefficients measured with FITC-dextran 4000. Furthermore, the cultivation period for cell differentiation could be significantly reduced by the subsequent dynamic cultivation.

Although these results are promising, future studies are required to analyze the integrity of the cell barrier in terms of tight junction formation and expression of enzymes and efflux transporters like P-glycoprotein.

To establish a platform for drug permeation studies to become a substitute for animal testing, cultivation parameters need also to be refined to enhance a synchronized ciliary beat in order to generate a functional mucociliary clearance. Therefore, challenges in designing a physiological cellular architecture and configuring a dynamic microenvironment to mimic the nasal mucosa have to be overcome. In the future, this platform could also be expanded to include additional tissues, such as the olfactory epithelium, to study more complex transport mechanisms, such as the nose-to-brain delivery.

## Figures and Tables

**Table 1:** The TEER values for the ANaMuc model obtained after 7, 10 and 14 days from measurements in the NM-chip after transfer and, for reference, in the Transwell^®^ Insert under static conditions.

ALI Cultivation [Days]	NM-Chip TEER [Ω∙cm^2^]	Transwell^®^ Insert TEER [Ω∙cm^2^]
7 days	430 ^b^	293 ± 16 ^a,d^
10 days	327 ^b^	189 ± 14 ^a^
14 days	556 ± 72 ^c^	412 ± 39 ^a^

Ref. ^a^ [30], ^b^ n = 1, ^c^ Mean ± SD, n = 3, ^d^ data was only available for 6 days.

## Data Availability

Data are available upon reasonable request from the corresponding author.

## Supplementary Materials

The following supporting information can be downloaded at zenodo.org: https://doi.org/10.5281/zenodo.8181106, Video S1: ANaMuc in NM-Chip 7d-3h-video, Video S2: ANaMuc in NM-Chip 10d-3h-video, Video S3: ANaMuc in NM-Chip 14d-3h-video, Video S4: Membrane Transfer NM-chip_video.
